# Formation of Unstable and very Reactive Chemical Species Catalyzed by Metalloenzymes: A Mechanistic Overview

**DOI:** 10.3390/molecules24132462

**Published:** 2019-07-04

**Authors:** Henrique S. Fernandes, Carla S. Silva Teixeira, Sérgio F. Sousa, Nuno M. F. S. A. Cerqueira

**Affiliations:** UCIBIO@REQUIMTE, BioSIM, Departamento de Biomedicina, Faculdade de Medicina da Universidade do Porto, Alameda Professor Hernâni Monteiro, 4200-319 Porto, Portugal

**Keywords:** metalloenzymes, transition metals, catalytic mechanism, iron, zinc, molybdenum

## Abstract

Nature has tailored a wide range of metalloenzymes that play a vast array of functions in all living organisms and from which their survival and evolution depends on. These enzymes catalyze some of the most important biological processes in nature, such as photosynthesis, respiration, water oxidation, molecular oxygen reduction, and nitrogen fixation. They are also among the most proficient catalysts in terms of their activity, selectivity, and ability to operate at mild conditions of temperature, pH, and pressure. In the absence of these enzymes, these reactions would proceed very slowly, if at all, suggesting that these enzymes made the way for the emergence of life as we know today. In this review, the structure and catalytic mechanism of a selection of diverse metalloenzymes that are involved in the production of highly reactive and unstable species, such as hydroxide anions, hydrides, radical species, and superoxide molecules are analyzed. The formation of such reaction intermediates is very difficult to occur under biological conditions and only a rationalized selection of a particular metal ion, coordinated to a very specific group of ligands, and immersed in specific proteins allows these reactions to proceed. Interestingly, different metal coordination spheres can be used to produce the same reactive and unstable species, although through a different chemistry. A selection of hand-picked examples of different metalloenzymes illustrating this diversity is provided and the participation of different metal ions in similar reactions (but involving different mechanism) is discussed.

## 1. Introduction

Nature has tailored a wide range of proteins that play a vast array of functions in all living organisms and from which their survival and evolution depends on [1]. Some of these proteins require the presence of metal cofactors that can be as small as a metal ion or as large as a heterocyclic organometallic complex [2,3,4,5]. These proteins are called metalloproteins and they are estimated to account for almost one-third of the full proteomic world [6,7].

Metalloproteins can play many different roles in nature, such as catalytic mediators, transport, storage, and signal transduction [6,7,8,9,10]. Among the most important ones are those with enzymatic activity, commonly named metalloenzymes [11,12,13,14]. These enzymes catalyze some of the most important biological processes in nature, such as photosynthesis [15,16], respiration [17,18], water oxidation [19], molecular oxygen reduction [20,21], and nitrogen fixation [22]. They are also among the most proficient catalysts in terms of their activity, selectivity, and ability to operate at mild conditions of temperature, pH, and pressure [23]. In the absence of these enzymes, these reactions would proceed very slowly, if at all, suggesting that these enzymes made the way for the emergence of life as we know today.

Over the years, a wealth of knowledge on the metal-dependent enzymes has been accumulated, including data from X-ray crystallography, kinetic and biochemical properties, and reaction mechanisms. These studies revealed a variety of metal cofactors [24] that catalyze very specialized chemical reactions [25]. The majority of these cofactors contain first-row transition-metal ions, such as manganese [26], iron [27], cobalt [28], copper [29], or zinc [30]; the alkaline earth metal magnesium [31] or even second-row and third-row transition metal ions, such as molybdenum [32] and tungsten [32,33], respectively. Most of these metal ions are usually coordinated to the proteins by imidazole substituents in histidine residues [34], thiolate substituents in cysteine residues [35], and carboxylate groups provided by aspartate or glutamate residues. Other metal ions can also coordinate to organic cofactors, such as the tetradentate N4 macrocyclic ligands present in hemes, pyranopterin, and among others. The nature and the charge of the ligands in the coordination sphere of the metal ion dictates its coordination numbers and/or geometries and ultimately defines its role in the biological systems. For example, in the case of Zn^2+^, the ion can play either a catalytic, co-catalytic, or structural role in the enzyme [36,37].

The role played by the protein in catalysis of the metalloenzymes is often disregarded. However, this is far from being correct, since proteins also have an important role in the reactivity of the metal cofactor [23]. For example, they can hamper a symmetrical binding of the substrate to a metal ion favoring by this way a stereoselective reaction; they can prevent the direct interaction between a substrate and the metal ion, through the coordination of several amino acid residues to the metal ion, etc. All of these facts together indicate that the chemistry played by metalloenzymes is not exclusively dependent on the metal ion, but instead there is a reciprocal interaction between the metal and the protein.

The chemistry catalyzed by metalloenzymes is very specific from enzyme to enzyme and commonly involves the formation and/or sequestration of extremely unstable chemical species that are ultimately transferred to a given substrate [9,38]. In the majority of the cases, once the product of the reaction is released to the solvent, the metal cofactor needs to be recycled back to its original reactive form and only afterwards the enzymatic turnover takes place.

In this review, the catalytic mechanism of a diverse selection of metalloenzymes will be reviewed. All the included examples describe several enzymes in which the cofactors contain one or more metal ions that can play a catalytic or co-catalytic function and involve the formation of unstable and very reactive reaction intermediates such as hydroxide anions (OH^−^), hydrides (H^−^), radicals, and superoxide molecules.

## 2. Enzymes that Catalyze the Formation of Hydroxide Anions

Hydroxide is a diatomic anion, which consists of an oxygen and a hydrogen atom held together by a covalent bond that carries a negative charge [39]. It is a strong nucleophile that is employed by a variety of metalloenzymes to catalyze hydrolysis or hydration reactions. The formation of such a highly reactive and unstable anion is not a straightforward process (especially under physiological conditions) and can only be achieved by a reduced number of enzymes that requires the presence of special metal cofactors. These often include zinc in their structure, but occasionally, other transition metals, such as manganese and nickel, can also be found.

In these reactions, the metal cofactor always acts as an electron pair acceptor (as a metal-based Lewis acid) that stabilizes the highly reactive hydroxide anion, thereby ensuring that an activated nucleophile is available for catalysis at physiological pH. Although these metalloenzymes seem to share a similar function in nature, the chemistry that they catalyze is different. In this section, we compare different metal coordination spheres present on enzymes that catalyze the formation of hydroxide anions, their particularities, and the mechanism that allows these enzymes with such different metals and coordination spheres to carry out similar reactions, while employing a diverse chemistry.

### 2.1. Mono-nuclear Zinc Cofactor

α-Carbonic anhydrase (CA, EC 4.2.1.1) belongs to a family of enzymes that catalyze the inter-conversion of carbon dioxide (CO_2_) to carbonic acid (H_2_CO_3_) though the formation of a hydroxide ion (bound to zinc) at neutral pH [40,41,42]. In this particular case, a tetracoordinate Zn ion is responsible for the formation of hydroxide intermediate that triggers the conversion of carbon dioxide into carbonic acid.

**Scheme 1:**
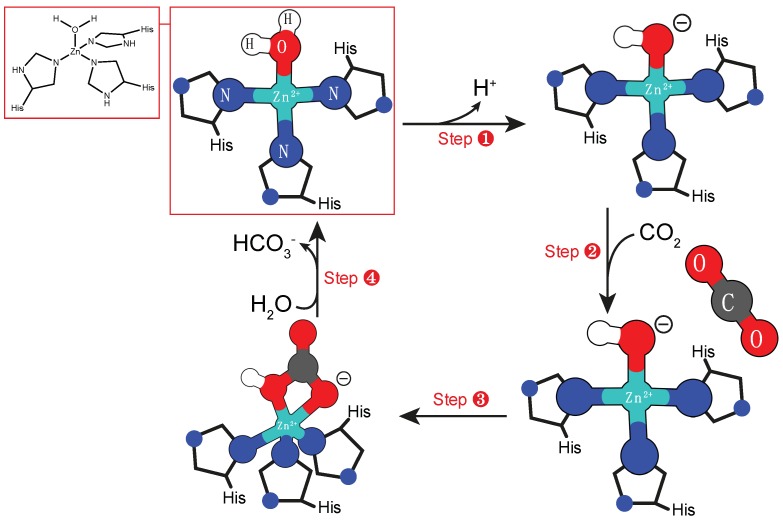
General description of the catalytic mechanism of α-Carbonic anhydrase (CA). Only the atoms involved in the first coordination shell of the metal ion were included to simplify the representation.

The mechanism starts with a water molecule bonded directly to the Zn(II) cofactor that is coordinated to three histidine residues [43]. This complex polarizes the bonds between the oxygen and the two hydrogen atoms from the water molecule, making these chemical bonds weaker, and the oxygen atom slightly more positive. This effect favors the proton transfer from the water molecule to a neighboring histidine residue originating from a hydroxide anion that remains coordinated to the Zn(II) ion (Scheme 1—Step ❶). When carbon dioxide becomes available in the active site (Scheme 1—Step ❷), the nucleophilic oxygen from the hydroxide ion attacks the carbon from carbon dioxide, at the same time that one of the oxygen atoms from the same molecule binds directly to the Zn(II) cofactor (Scheme 1—Step ❸). These chemical reactions induce a conformational rearrangement on the metal cofactor that goes from a distorted tetrahedral conformation to a trigonal bipyramidal configuration. This new rearrangement turns carbon dioxide very close to the hydroxide ion (both of them coordinated directly to the Zn(II) ion) favoring by this way the reaction between both ligands. At the end of the reaction, a water molecule becomes coordinated to the Zn ion forming a pentacoordinate complex that triggers the release of the bicarbonate molecule, making the enzyme ready for a new catalytic cycle (Scheme 1—Step ❹) [41,44,45,46,47,48,49].

CA has been the focus of many experimental [50,51,52,53,54,55,56,57,58,59,60] and theoretical [61,62,63,64,65,66,67,68,69,70,71,72,73,74,75,76,77] investigations, many of them devoted to the study of its catalytic mechanism. Those studies show values of *k*_cat_ in the range of 10^4^ to 10^6^ s^−1^ [78] and suggests that the rate-limiting step of the reaction mechanism is the deprotonation of metal bound water molecules [79], although this hypothesis is not consensual [71].

Histone Deacetylase 8 (HDAC8, EC 3.5.1.98) catalyzes the deacetylation of lysine residues on the N-terminal part of the core histones [80,81]. Similar to CA, the reaction catalyzed by HDAC8 involves a zinc cofactor that reacts with a water molecule and from which results a hydroxide anion. This reaction intermediate is a sturdy nucleophile that attacks the substrate directly from the metal ion. In spite of the similarities between the mechanisms of HDAC8 and CA, these enzymes have different amino acid residues coordinated to the Zn(II) ion and follow different mechanisms.

In CA, the Zn(II) ion is coordinated with three histidine residues, whereas in HDAC8, the Zn(II) ion is coordinated to one histidine and two aspartate residues.

**Scheme 2:**
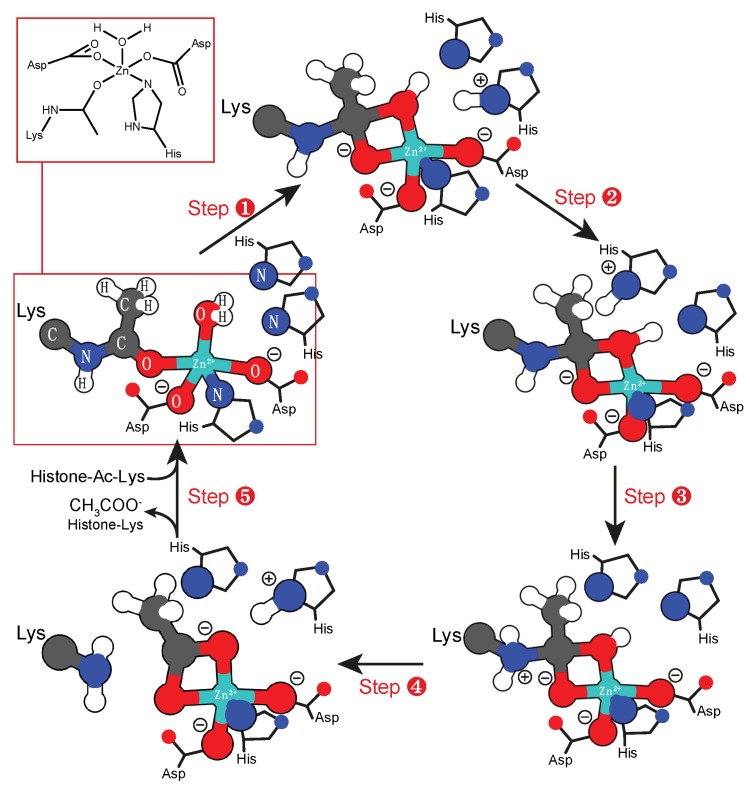
General description of the catalytic mechanism of Histone Deacetylase 8 (HDAC8). Only the atoms involved in the first coordination shell of the metal ion were represented to simplify the representation.

In HDAC8, the catalytic mechanism begins with the binding of a carbonyl group of an acetylated lysine to the Zn(II) ion that is tetra-coordinated with three amino acid residues and a water molecule. At the end of this process, a penta-coordinated complex is obtained. Only afterwards, the formation of the hydroxide anion takes place. This reaction occurs by the abstraction of a proton from the water molecule by a neighboring histidine residue, which is concomitant with the formation of a covalent bond between the oxygen from the hydroxide anion and the sp2 carbon from the acetyl group (Scheme 2—Step ❶). The next step involves a proton exchange between the two active site histidine residues, through the oxygen from the hydroxide anion (Scheme 2—Step ❷). The protonated histidine then donates a proton to the nitrogen atom of the substrate (Scheme 2—Step ❸). The last step of the mechanism involves the formation of an acetyl group and the release of the lysine residue from histones (Scheme 2—Step ❹). The enzymatic turnover is obtained when the Zn(II) cofactor reacts again with a new water molecule, making the enzyme ready for a new cycle (Scheme 2—Step ❺) [82,83].

Computational studies have shown that the limiting step of the catalytic mechanism is the first one with a calculated overall activation free energy barrier of 18.3 ± 0.4 kcal/mol [84], which is consistent with the experimental value of 17.7 kcal/mol that can be estimated from the experimental *k_cat_* value of 0.90 ± 0.03 s^−1^ for HDAC8 [85].

The differences that are observed between the catalytic mechanisms of CA and HDAC8 are considered to be determined by the nucleophilic strength of the hydroxide anion that is bonded to the Zn(II) cofactor. This is modulated by the capacity of the Zn(II) ion to behave as an electron pair acceptor (metal-based Lewis acid) that, in the case of these two enzymes, is enhanced by the enzyme through the coordination of different amino acid residues to the zinc ion. HDAC8 has two aspartate residues and one histidine residue coordinated to Zn(II) instead of the three histidine residues that are bonded to Zn(II) in CA. This means that HDAC8 has a higher capacity to shelter the negative charge from the hydroxide anion than CA, turning by this way its hydroxide anion into a worse nucleophile [86,87,88]. Although this would suggest that the hydroxide anion in HDAC8 would be less reactive and therefore the enzyme less efficient than CA, this is not observed. In fact, this feature seems to be endorsed by the enzyme due to the different substrates that both enzymes catalyze. In the case of HDAC8, the protonation of the substrate is required, which is only possible because the proton from the hydroxide anion can be easily interchanged with the active site histidine residues. This step would not be feasible if the hydroxide anion was very reactive, as it occurs in the mechanism of the other enzyme. In the case of CA, this step is not required and, therefore, the performance of the enzyme is improved with a more reactive hydroxide anion.

### 2.2. Binuclear Manganese Cofactor

Prolidase or proline dipeptidase (EC 3.4.13.9) is a binuclear metallopeptidase that hydrolyzes dipeptides with proline or hydroxyproline at the carboxyl terminus [89,90]. These binuclear metalloenzymes hydrolyze mainly Gly-Pro dipeptides but they are also active against Ala-Pro, Met-Pro, Phe-Pro, Leu-Pro, and Val-Pro [89,91,92].

The X-ray structures of human prolidase indicates the presence of two catalytic Mn(II) ions in the active site that are hexa-coordinated to a histidine, two glutamate, two aspartate residues, and a water molecule [93,94]. Both Mn(II) ions are bridged between themselves by a hydroxide anion and two *syn*-bidentate carboxylate groups from the aspartate and glutamate residues. Due to this disposition, the Mn1 ion has a distorted octahedral configuration, whereas the Mn2 ion adopts a trigonal bipyramidal configuration.

**Scheme 3:**
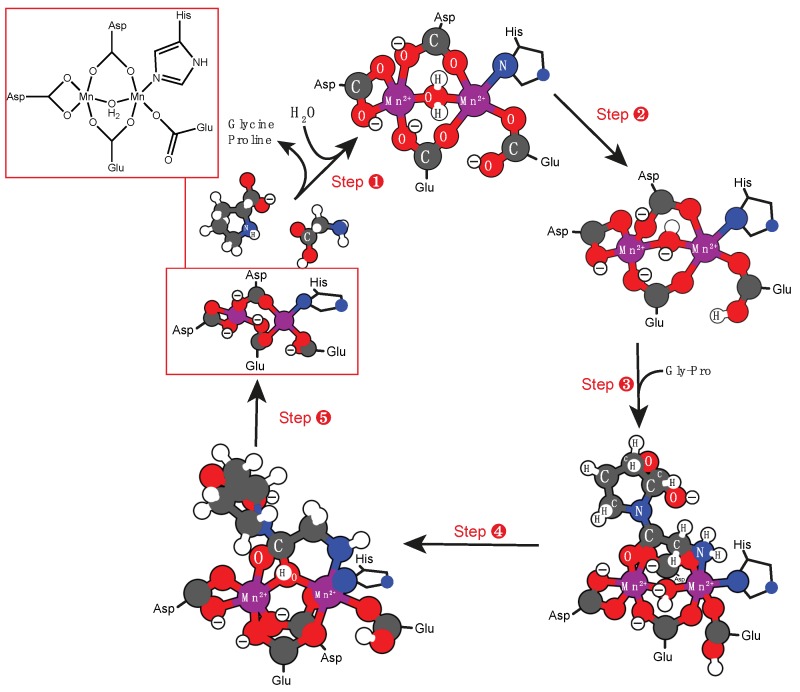
General description of the catalytic mechanism of prolidase. Only the atoms involved in the first coordination shell of the metal ion were represented to simplify the representation.

The catalytic mechanism starts with the activation of the Mn1–Mn2 metal ions bridging one water molecule (Scheme 3—Step ❶). This is catalyzed by the carboxylate group of one of the neighbor glutamate residues, which abstracts a proton from the water molecule, and from which results a hydroxide anion that becomes coordinated to both metal ions (Scheme 3—Step ❷).

When the substrate is available in the active site (for example, Gly-Pro), it becomes coordinated to both Mn(II) ions and it is stabilized by a network of hydrogen bonds created by one neighbor arginine, two histidine residues, and two aspartate residues that are coordinated to both metal ions (Scheme 3—Step ❸). The formation of this complex endorses the polarization of the peptide bond of the substrate that favors the nucleophilic attack of the hydroxide anion to the partially positively charged (δ^+^) carbonyl group of the substrate (Scheme 3—Step ❹). Once this step is completed, a tetrahedral reaction intermediate is obtained. The next step of the catalytic mechanism is proposed to occur in two simultaneous reactions: (i) The proton transfer from the hydroxide anion to the nitrogen atom enclosed on the proline ring with the help of a neighbor glutamate residue and (ii) the cleavage of the peptide bond of the substrate (in this case, Pro-Gly) (Scheme 3—Step ❺). [89] Once this step is finished, Gly and Pro leave the active site and a water molecule from the solvent binds to the bimetallic center. There, it is activated by a glutamate residue that assists a proton abstraction, regenerating the enzyme for a new catalytic cycle (Scheme 3—Step ❶) [93,95].

Experimental data show a *k*_cat_ of 683 s^−1^ for the human prolidase catalyzing the substrate Gly-Pro [96].

### 2.3. Binuclear Nickel Cofactor

Urease (EC 3.5.1.5), in terms of function, belongs to a family amidohydrolases and phosphotriesterases [97] that employs two asymmetric Ni(II) metal ions in the active site to degrade urea into carbon dioxide and ammonia [98,99]. The active site also has an unusual carbamylated lysine (Lys*) residue that establishes a bridge between both catalytic Ni(II) ions.

One of the Ni(II) cations, Ni1, is penta-coordinated to an oxygen from the lysine residue, a hydroxide anion, a water molecule, and two histidine residues. The second Ni(II), Ni2, is hexa-coordinated to the other oxygen atom of the lysine, to the same hydroxide anion, a water molecule, two histidine residues, and an additional aspartate residue.

Similar to what is observed in all the other enzymes discussed in this section, the hydroxide anion is rapidly formed through the reaction of a water molecule with the metal cofactor that loses a proton to an active site residue.

**Scheme 4:**
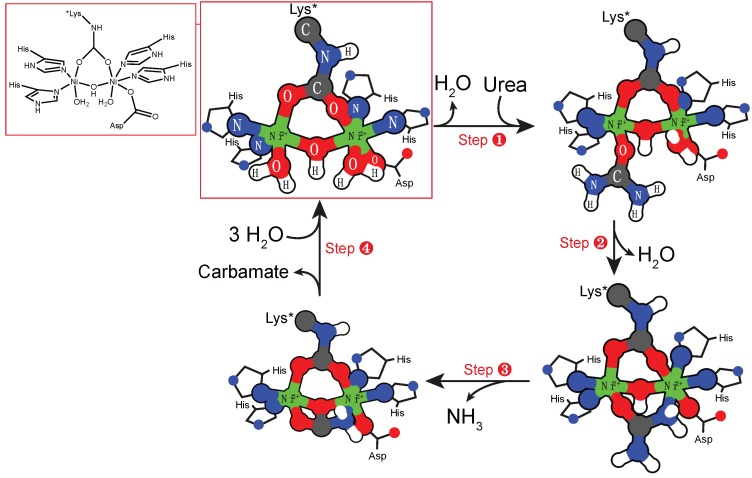
General description of the catalytic mechanism of urease. Only the atoms involved in the first coordination shell of the metal ion were represented to simplify the representation.

Once the substrate, urea, is available in the active site, the water molecules coordinated to the Ni1 ion dissociate from the metal cofactor and urea becomes coordinated directly to the Ni1 ion through the oxygen atom (Scheme 4—Step ❶). Afterwards, the water molecule coordinated to Ni2 ion also dissociates from the active site and the amino group from the bonded substrate also becomes coordinated to the Ni2 ion (Scheme 4—Step ❷). This configuration enhances the proximity between urea and the hydroxide ion that is also bidentate to both Ni(II) ions. In the next step, the nucleophilic attack of the hydroxide anion to the carbon atom of urea occurs, at the same time that one proton is transferred from the first molecule to the amino group of the second one. From this reaction results a carbamate molecule that remains coordinated to the metal cofactor and ammonia that is released to the solvent (Scheme 4—Step ❸). The generation of the active site occurs after the dissociation of carbamate from the metal cofactor and, the subsequent coordination of three water molecules directly to the Ni(II) ions (Scheme 4—Step ❹). In solution, the carbamate molecule is spontaneously degraded into carbon dioxide and ammonia [100,101].

Experimental studies indicates a *k*_cat_ for the substrate urease that can vary between 1250 and 3500 s^−1^ depending on the organism and the temperature and pressure conditions [78,102,103,104]. Based on a kinetic analysis, it has been predicted that urease is the most proficient enzyme identified to date to convert urea into carbon dioxide [104].

### 2.4. Binuclear Zn Cofactor

So far, we have discussed the catalytic mechanism of metalloenzymes that catalyze the formation of hydroxide anions and employ a monodentate Zn cofactor (CA and HDAC8) or a binuclear Mn (prolidase) or binuclear Ni (urease) cofactors. The main difference between the mechanisms employed by these enzymes is the way that each substrate reacts with the hydroxide anion. In the case of Ni(II)- and Mn(II)-dependent enzymes, the substrate become bidentate to two metal ions becoming stereo-chemically positioned to undertake the nucleophilic attack of the coordinated hydroxide anion. In the case of the monodentate Zn enzymes, this is not observed since the substrate becomes bidentate to the same Zn ion that also shelters the same hydroxide anion. This happens because Zn is an exceptionally good Lewis acid for biological catalysis and allows a fast interconversion among its four-, five-, and six-coordinate states.

Although these findings suggested that the Zn-dependent metalloenzymes that are involved in the formation of hydroxide anions are all monodentate, one enzyme was found that produces the same unstable molecule but lodges two catalytic Zn ions in the active site. This enzyme is leucine aminopeptidase (LeuAP; EC 3.4.11.1) and catalyzes the hydrolysis of leucine residues from the *N*-terminus of a wide variety of di- and tripeptide substrates [105,106,107,108,109,110].

LeuAP, together with other aminopeptidases, carboxypeptidases, phosphatases, β-lactamase, and others, belongs to the hydrolase enzyme class [111,112]. The zinc hydrolase superfamily includes both mono zinc enzymes, such as carboxypeptidase or thermolysin, as enzymes that harbors co-catalytic zinc sites, such as, aminopeptidase, β-lactamase, or alkaline phosphatase [113].

The active site of this enzyme, LeuAP, contains two non-equivalent Zn ions placed at about 3.02 Å away from each other [107]. Each metal ion has a different coordination sphere that endorses different characteristics to each metal ion. The Zn(II) ion at site one (Zn1) is penta-coordinated to two aspartates and one glutamate residue. The Zn(II) ion at site two (Zn2) is also penta-coordinated with two aspartates, one glutamate, and one lysine. The glutamate and one of the aspartates are also bridging the two metal ions [114].

**Scheme 5:**
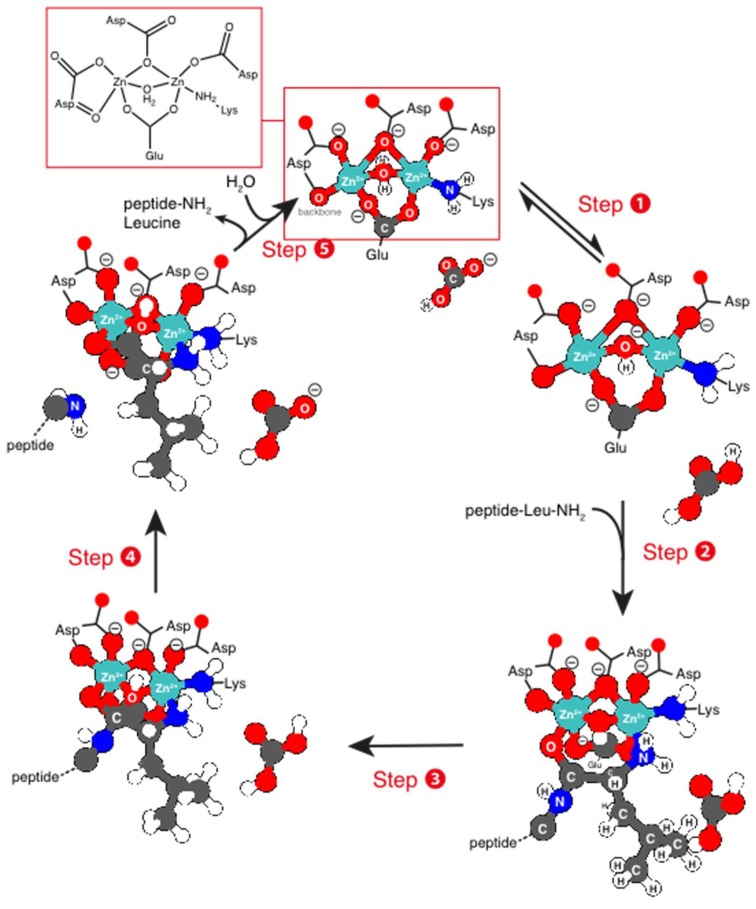
General description of the catalytic mechanism of leucine aminopeptidase (LeuAP). Regarding amino acid residues belonging to the protein, only the atoms involved in the coordination of the metal were represented to simplify the representation.

The currently accepted mechanism for this enzyme was proposed based on computational means and agrees very well with the available experimental data [114,115]. The first step of the catalytic mechanism starts by the activation of the Zn1–Zn2 metal ions through the abstraction of a proton from the water molecule (that is coordinated to both ions) by carbonate and from which results a hydroxide anion (Scheme 5—Step ❶). Then, the peptide (substrate) becomes coordinated to the metal ion through the carbonyl group of the backbone and the terminal amine group (Scheme 5—Step ❷). Afterwards, the nucleophilic attack of the hydroxide anion to the α-carbon of the scissile peptide bond of the substrate to form a tetrahedral gem-diolate reaction intermediate occurs (Scheme 5—Step ❸). Subsequently, the carbonic acid donates its previously acquired proton to the nitrogen atom of the scissile peptide bond of the substrate, at the same time that Asp273 accepts a proton from hydroxyl group attached to α-carbon of the substrate (Scheme 5—Step ❹) [108,114,115]. These concerted proton transfers lead to the cleavage of the peptide bond at the *N*-terminus of the peptide (preferentially) releasing a leucine molecule (Scheme 5—Step ❺).

A computational study has shown that the rate-limiting step for the reaction with l-leucine-*p*-nitroanilide is the cleavage of the peptide bond with a barrier of 17.8 kcal/mol [114]. This result is in agreement with the experimentally measured barrier of 18.7 kcal/mol estimated from the *k_cat_* value of 0.117s^−1^ [116].

Comparing the mechanism of the mononuclear Zn-dependent enzymes with the binuclear ones, they present one big difference. In the first type of enzymes, the substrate becomes coordinated to the same Zn ion that also shelters the same hydroxide anion. In the binuclear Zn(II) cofactors, the substrate acts as bidentate ligand, coordinating both metal ions and promoting a close and very specific access of the hydroxide anion (also coordinated to both metal ions) to the electrophilic atom of the substrate.

The metal site of LeuAP is commonly termed as a readily exchangeable site in which the Zn1 ion can be replaced by Mg^2+^, Co^2+^, and Mn^2+^ ions without destroying the activity of the enzyme. Site two, however, is referred to act as a tight binding site, where the Zn2 ion can only be replaced by a Co^2+^ ion [117]. Computational studies have shown that the electronic nature of the metal ion at site one influences only the generation of the gem-diolate intermediate, suggesting that different metals can be used to turn the reaction specific for different types of substrate [114].

## 3. Enzymes that Catalyze the Hydride Transfer Reactions

Hydride transfer is another elementary process catalyzed by several metalloenzymes. Unlike hydroxide anions (OH^−^), hydride ions do not exist in aqueous solutions as free ions. For this reason, they can only be transferred directly from one organic moiety to another one through the simultaneous breakage and formation of covalent bonds. The metal cofactor present in the enzymes that catalyze this type of reaction often provides the binding site of the substrate and are directly involved in the hydride transfer. The metalloenzymes that house molybdenum, tungsten, and zinc ions often catalyze hydride transfer reactions. The catalytic mechanism of these enzymes will be reviewed in the next sections.

### 3.1. Molybdenum and Tungsten Cofactors

The molybdenum is found in a variety of enzymes that catalyze hydride transfers that are coupled with redox reactions. Examples of these enzymes are present in the xanthine oxidase (EC 1.17.3.2, XO) [118], DMSO reductase (EC 1.8.5.3) [119], and sulfite oxidase (EC 1.8.3.1, SO) [120] families [121]. In these enzymes, the Mo ion is coordinated to one or two pyranopterin dithiolene ligands (PDT), forming what is known as a molybdenopterin cofactor. The enzymes from DMSO reductase are coordinated to two PDT ligands, while the enzymes from the XO and SO families have only one PDT ligand. Depending on the enzyme, the coordination sphere around the Mo ion is completed by a wide range of ligands that can range from oxygen or sulfur anions, or side chains from amino acid residues such as cysteine, selenocysteine, glutamates, or serine [122]. In prokaryotes, tungsten can also be found in the place of Mo, but they share similar functions [123].

The essential role played by Mo (or W) during catalysis is to promote a controlled *oxo*-transfer between the substrate and the metal cofactor that involves, at some point, a hydride transfer that is coupled to an electron transfer between the substrate and the external oxidant, reductant species. 

The representative reactions that are catalyzed by these enzymes are generally well understood and can be summarized based on the following chemical equations:

- Xanthine oxidase:  RCHO + H_2_O    →   RCO_2_H + 2 H^+^ + 2 e^−^;
- Sulfite oxidase:    SO_3_^−2^ + H_2_O   →  O_4_^−2^ + 2 H^+^ + 2 e^−^;
- DMSO reductase:   HCO_2_^−^       →   O_2_ + 2 H^+^ + 2 e^−^.

**Scheme 6:**
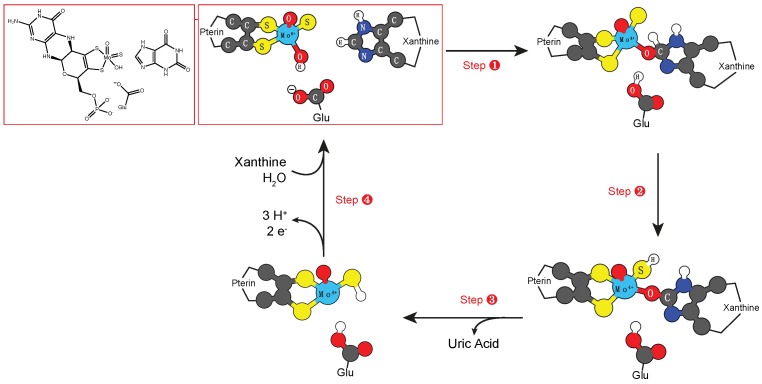
General description of the catalytic mechanism of xanthine oxidase (XO). Regarding amino acid residues belonging to the protein, only the atoms involved in the coordination of the metal were represented to simplify the representation.

In the case of the enzymes from the XO family, the first step of the catalytic mechanism involves a proton abstraction from the equatorial Mo–OH, by a glutamate residue [95,124]. Subsequently, the nucleophilic attack of the oxygen that is coordinated to the Mo ion to a carbon of the substrate occurs (Scheme 6—Step ❶). This leads to the formation of a penta-coordinated complex around the Mo ion. Afterwards, the hydride transfer from the substrate to the Mo = S group occurs (Scheme 6—Step ❷), as does the cleavage of the bond between the oxygen and the Mo ion (Scheme 6—Step ❸). After the oxidation of the cofactor (electron transfer from the Mo to the other redox-active centers present on the enzyme), a water molecule occupies the free coordination position around the Mo ion. The enzymatic turnover only occurs after two deprotonation steps and the enzyme is ready for a new cycle (Scheme 6—Step ❹).

The *k_cat_* for the conversion of xanthine to uric acid by the human XO is of 18.3 s^−1^ [125] but it can vary between 0.41 and 108 depending on the species and the experimental conditions [78].

The enzymes from the SO family also use a water molecule to catalyze a simple *oxo*-transfer from sulfite to form sulfate and the mechanism also involves a hydride transfer from the substrate to the metal cofactor. These enzymes thus employ a similar mechanism to the one that was described for the enzymes of XO family [126].

The catalytic mechanism of the enzymes from the DMSO family is quite different from the ones present on the SO and XO families. Part of these differences may be attributed to the presence of two PDT ligands in the cofactor instead of one, and of course by the different substrates that they catalyze (they are negatively charged) [127,128].

The catalytic mechanism of formate dehydrogenase (EC 1.2.1.2, FdH) is described below to exemplify how hydride atom transfer reactions can be catalyzed by the enzymes from the DMSO family [129,130].

FdH catalyzes the oxidation of the formate anion to carbon dioxide in a redox reaction that involves the transfer of a hydride and two electrons from the substrate to the external oxidizing species [131].

**Scheme 7:**
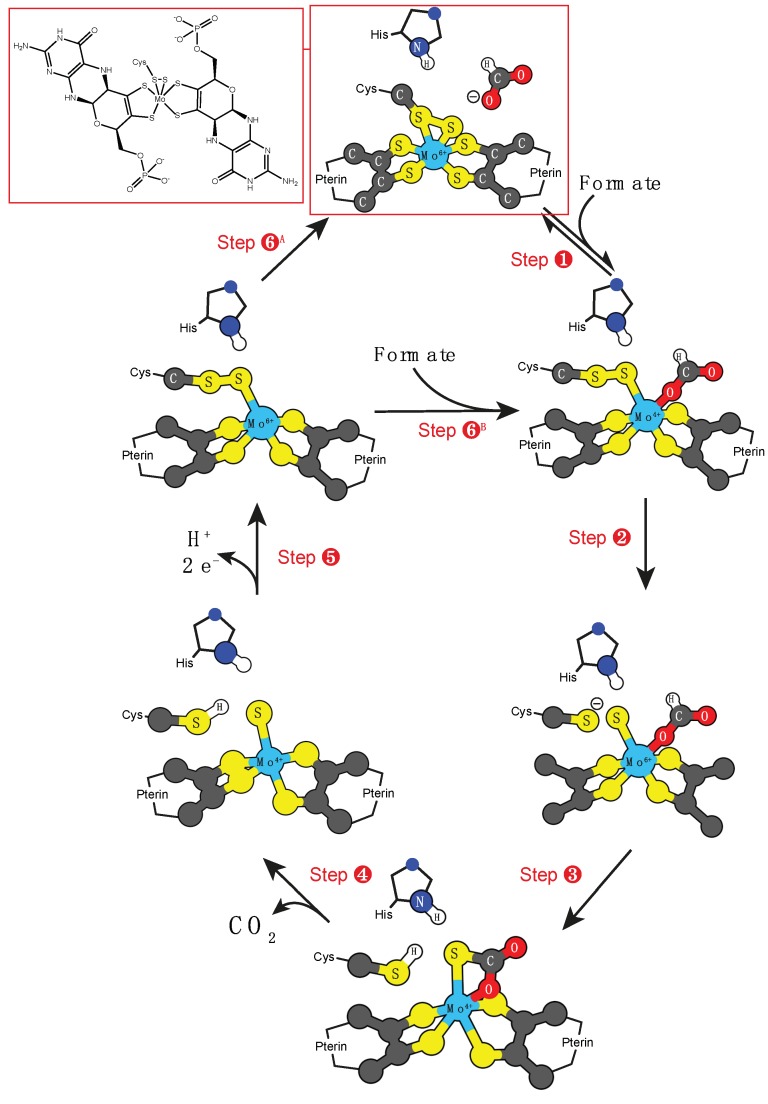
General description of the catalytic mechanism of formate dehydrogenase (FdH). Regarding amino acid residues belonging to the protein, only the atoms involved in the coordination of the metal were represented to simplify the representation.

When substrate is not present at the active site of FdH, the Mo (VI) ion is hexa-coordinated and no free position is available to bind the substrate (formate) (Scheme 7—Step ❻**^A^**). In such state, the cofactor has a total charge of −1 and this led several authors to propose, in the past, that the catalytic mechanism should occur through the second shell of the metal ion, as the approach and binding of the negative charged substrate to the also negatively charged cofactor would be extremely unfavorable. Recently, it was found that the mechanism takes place through a first-shell type of mechanism, in which the substrate binds and reacts directly with the Mo ion. This is only possible due to a rearrangement on the coordination sphere around the Mo ion that takes place when the substrate comes close to it. This rearrangement was named “sulfur-shift mechanism” and is defined as a change in the Mo ion coordination sphere, which involves a first-to-second shell displacement (shift) of the SeCys ligand of the Mo ion (Scheme 7—Step ❻**^B^**), resulting in a free coordination position that is used by the enzyme to coordinate an oxygen atom from formate directly to the Mo ion (Scheme 7—Step ❻**^B^** or ❶) [132]. The mechanism was first discovered by theoretical means and later on validated by experimental means [132,133,134,135]. Interestingly, the same type of activation mechanism was found in another enzyme from the DMSO family (nitrate reductase) but catalyzes a different reaction.

The next step of the catalytic mechanism in FdH involves the cleavage of the bond that connects SeCys to the inorganic sulfur (Scheme 7—Step ❷). This cleavage is possible thanks to the side chain of an active site histidine residue, which forms a hydrogen bond with the resulting selenol anion and stabilizes it. Subsequently, the active site histidine residue catalyzes the proton transfer from formate to SeCys by forming a hydrogen bond with the selenol (Scheme 7—Step ❸). Once this reaction is complete, the SeCys moves away from the active site, resembling what has been observed in the re-interpretation of the X-ray data of reduced from Fdh-H [136]. At the same time that the proton transfer between formate and the SeCys occurs, a pair of electrons is transferred from the carbon to the cofactor that becomes reduced.

A great deal of discussion has been held on this step, since this step has always been regarded as a hydride transfer and not a proton transfer. A classical hydride transfer involves the transfer of a hydrogen atom and a pair of electrons from the carbon of the substrate to another molecule. In the case of FdH, a similar mechanism occurs. Indeed, there is a proton transfer between the substrate and the SeCys, and a pair of electrons are transferred from the carbon of formate to the metal cofactor.

Once the “hydride transfer” takes place, the resulting carbon dioxide molecule does not dissociate from the cofactor and remains bound to the Mo ion. The next step of the catalytic cycle involves the release of carbon dioxide (Scheme 7—Step ❹) and the oxidation of the cofactor (Scheme 7—Step ❺). The enzymatic turnover only takes place once the proton bound to the SeCys side chain is transferred to the solvent or to another residue in the catalytic pocket. This event forces a conformational rearrangement of the loop where SeCys is located, to occupy a position near the Mo ion. Once this is accomplished, the SeCys forms a covalent bond with the sulfide ligand that is coordinated to the Mo ion. From this point, two pathways can be followed. If no substrate is available at the active site, the sulfur-shift mechanism is reversed, and the metal site adopts the original resting conformation (Scheme 7—Step ❻**^A^**). If formate is available, it can occupy the free coordinating position at the Mo ion and the catalytic process continues, without further delay (Scheme 7—Step ❻**^B^**) [137].

Computational studies addressing FdH have shown that no differences are observed if Mo or W are used to model the reactions that were described, accentuating the similar chemical role played by both metal ions.

A study combining computational and experimental data showed that the rate-limiting step of the FdH catalyzed reaction involves the transfer of the proton from the substrate to the selenium with an activation barrier of 21.2 kcal/mol [131].

The experimental *k*_cat_ for some molybdenum-containing formate dehydrogenases varies between 1.4 and 347 s^−1^ [131,138].

### 3.2. Zinc Cofactors

Zinc cofactors can also catalyze hydride transfers. This type of reaction can, for example, be found in alcohol dehydrogenases (EC 1.1.1.1, ADH), a group of dehydrogenase enzymes that facilitates the interconversion between alcohols, ketones, and aldehydes through the reduction of nicotinamide derivatives NAD^+^ or NADP^+^ to the corresponding 1,4-dihydronicotinamides [139,140,141,142]. This role is particularly important in humans and other animals, to degrade alcohols that otherwise would be toxic for the cells.

Each monomer of ADH houses two Zn ions in the active site: Zn1 and Zn2, which act as structural and catalytic metal ions, respectively. One of the Zn ions (Zn1) is coordinated to four cysteine residues and acts as a structural metal, whereas the other Zn ion (Zn2) is coordinated to two cysteine residues, one histidine residue, and a water molecule. The water molecule also establishes a hydrogen bond with a catalytic dyad formed by a serine and histidine residues from the active site.

**Scheme 8:**
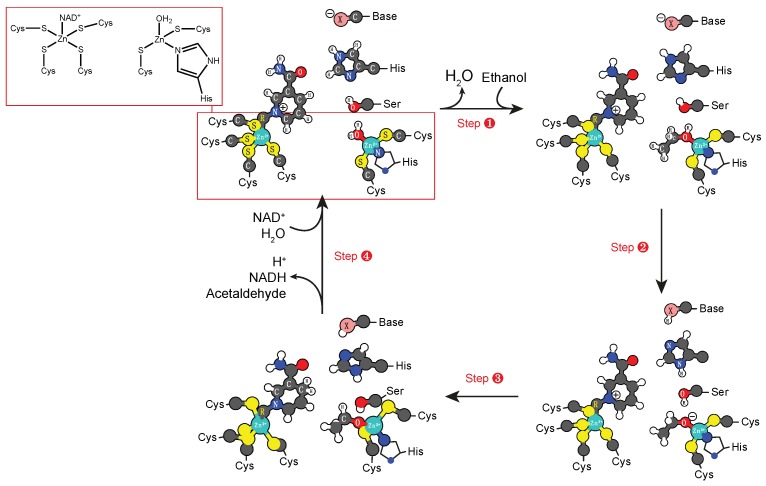
General description of the catalytic mechanism of alcohol dehydrogenases (ADH). Regarding amino acid residues belonging to the protein, only the atoms involved in the coordination of the metal were represented to simplify the representation.

The catalytic mechanism starts with the binding of NAD^+^ to the active site. [143] Once substrate (for example ethanol) is available, it becomes coordinated to the Zn2 ion and forces the water molecule to dissociate from the active site (Scheme 8—Step ❶). At the end of this process, the serine from the catalytic dyad becomes hydrogen bonded to the hydroxyl group of the substrate.

The next step involves the proton transfer from the hydroxyl group that is coordinated to the Zn(II) ion to an active site residue that behaves as a base. This occurs through a sequential proton transfer involving the serine and histidine residues from the catalytic dyad (Scheme 8—Step ❷). At the end of this step, an alkoxyde intermediate is generated that remains coordinated to the Zn2 ion. The third step of mechanism involves the hydride transfer, from the reaction intermediate to NAD^+^ (Scheme 8—Step ❸) [144]. From this reaction results the formation of an aldehyde that remains coordinated to the Zn(II) ion and NADH. The last step of the catalytic mechanism involves the dissociation of the aldehyde and the regeneration of the active site (Scheme 8—Step ❹). This is accomplished by the coordination of a water molecule to the Zn(II) ion, the proton transfer from the amino acid residue that behaves as a base with the catalytic dyad, and the loss of a proton [145].

In the full catalytic mechanism, only the Zn2 ion has a catalytic function, where it is important to polarize the C–O bond of the substrate, which is important to help the hydride migration to the NAD^+^ molecule. However, the importance of Zn1 ion in the mechanism cannot be underestimated. In fact, both metal ions have a preponderant role in the initial steps of the mechanism, where they help the correct orientation of the substrate in such an orientation that the reactive CH_2_ group of the nicotinamide ring of NAD^+^ points toward the hydroxyl group of the substrate.

Currently, it is still not clear which residue from the active site plays the function of the base and if it is in close contact with the catalytic dyad.

Computational studies predict an activation barrier of 21.9 kcal/mol for the rate-limiting step for the oxidation mechanism of benzyl alcohol by ADH [144].

Another recent theoretical study focused on the study of the reaction catalyzed by a secondary ADH showed, among others, a calculated overall barrier of 13.4 kcal/mol for (R)-2-butanol [143], which is consistent with the experimental value of 15 kcal/mol estimated from the experimental *k_cat_* value of 0.35 s^−1^ [146].

## 4. Enzymes that Catalyze the Formation of Radicals

The production of radical species by enzymes are of great importance. They produce very reactive and unstable chemical species that have a preponderant role in every living organism. Iron, manganese, and cobalt are the most common metal ions acting as cofactors present on these enzymes.

One of the best examples of enzymes that master the synthesis of radicals in nature are the ribonucleotide reductases (RNR). These enzymes catalyze the only pathway for de novo synthesis of the monomeric building blocks for both DNA replication and DNA repair [147,148,149].

Arguably the most difficult step during RNR-catalyzed ribonucleotide reduction is the initial activation of a chemically unreactive C–H bond [150,151]. The only pathway known to date involves the formation of a radical in a conserved cysteine residue that is capable of abstracting the 3′H-atom from the ribose moiety. This initial radical is, in turn, always generated via a metal-containing cofactor.

There are different RNRs that were divided in different classes based on the type of cofactor that they generate and require for the catalytic process [152]. The most studied ones are from class I and class II. Class I RNRs are oxygen-dependent enzymes and are present in both eukaryotes and eubacteria and contain a binuclear metal cluster formed by Fe or Mn [153,154,155]. Class II RNRs, found in both aerobic and anaerobic microbes, are oxygen-independent and use a cobalt-containing cobalamin (vitamin B12) cofactor [156,157].

### 4.1. Iron Cofactor

In the RNRs from class I, the tyrosyl radical is generated by a bi-iron metal cofactor that is channeled through 35 Å to a strictly conserved cysteine residue that is located in the active site very close to the substrate [158]. The active site and the metal cofactor are located in different subunits of the enzyme that need to dimerize in order to turn the enzyme active.

In the case of RNR from class Ia, the metal cofactor is composed by two Fe(II) ions that are coordinated to three glutamate and a histidine residue. Two of these glutamate residues are bi-dentated to both catalytic Fe(II) ions. Depending if one of these glutamates is bi-dentated or mono-dentated to the Fe(II) ions, the cofactor can adopt a five-coordinated or a distorted tetrahedral configuration [156,159].

**Scheme 9:**
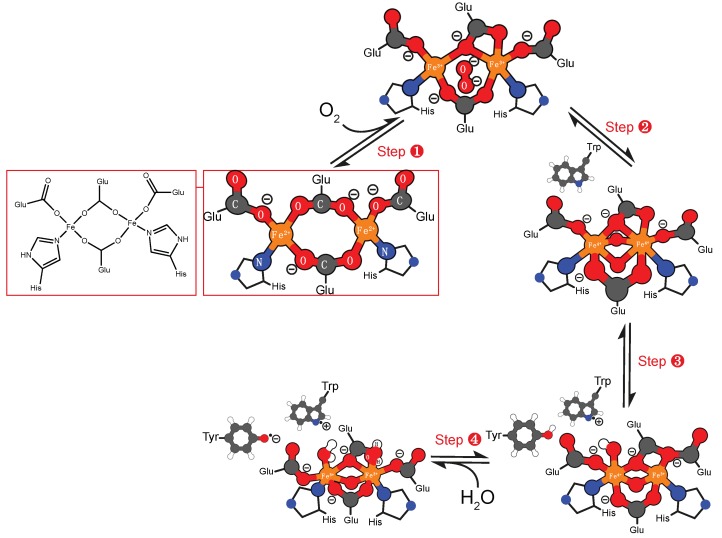
Schematic representation of the catalytic mechanism of ribonucleotide reductases (RNR) from class Ia (Fe–Fe). Regarding amino acid residues belonging to the protein, only the atoms involved in the coordination of the metal were represented to simplify the representation.

The radical begins being produced when a di-oxygen molecule reacts with the Fe(II)–Fe(II) metal cofactor, from which results a peroxide intermediate and an oxidized Fe(III)–Fe(III) metal cofactor (Scheme 9—Step ❶) [160]. During this process, one of the glutamate residues that was bi-dentated to both Fe ions forms a single oxo-bridge between the ions and only one of the Fe ions becomes bi-dentated.

Afterwards, the cleavage of the peroxide occurs, as does the formation of two oxo-bridges between the two metal ions, from which results the oxidation of Fe(III)–Fe(III) to Fe(IV)–Fe(IV) (Scheme 9—Step ❷). Subsequently, there is a homolytic cleavage of a hydrogen atom from a tryptophan residue to one of the oxygen atoms that is coordinated to the Fe(IV)–Fe(IV) metal cluster (Scheme 9—Step ❸). The resulting hydroxyl molecule dissociates from the cofactor, reducing one of the Fe(IV) to Fe(III). The free position in the coordination sphere around the iron ion is promptly filled by a water molecule that is available in the active site.

The final step of the mechanism involves the formation of the tyrosyl radical (Scheme 9—Step ❹). This involves the loss of a hydrogen atom from the other hydroxyl molecule. During this process, the hydroxyl is reduced to water and the Fe ion is reduced from Fe(IV) to Fe(III).

At the end of this reaction, a tyrosyl radical is generated, and the radical is transferred over 35 Å along the protein to the active site of the enzyme where it is finally accepted by a cysteine residue [156,161].

The experimental *k*_cat_ obtained for RNR from class Ia varies depending on the species, the concentration of an allosteric effector (ATP), and the quaternary structure of the enzyme used in the assays. They are, for example, in the range between 0.047 and 0.30 s^−1^ [162] or 2 and 10 s^−1^ [163] for the substrate cytidine diphosphate (CDP) and have values of 0.25 s^−1^ for uridine diphosphate (UDP), 0.18 s^−1^ for adenosine diphosphate (ADP), and 0.28 s^−1^ for guanosine diphosphate (GDP) [162].

### 4.2. Manganese Cofactor

In RNRs from class Ib, the tyrosyl radical is generated by a Mn–Mn cofactor, where both metal ions are catalytic. These enzymes also require the presence of an additional protein Nrd1, otherwise no activity is observed for the Mn(II)–Mn(II) center [154,164].

Little is known about the mechanism from which the radical is formed in this class of RNR enzymes. It is known that the process starts by the action of protein Nrd1 that reduces an O_2_ molecule to O_2_^−^ and tunnels it towards the Mn(II)–Mn(II) center of the enzyme. [164] The presence of protein Nrd1 is justified due to the limitation of Mn(II)–Mn(II) center that cannot reduce the oxygen, as it is observed in the Fe(II)–Fe(II) cluster of RNRs from class Ia [154].

**Scheme 10:**
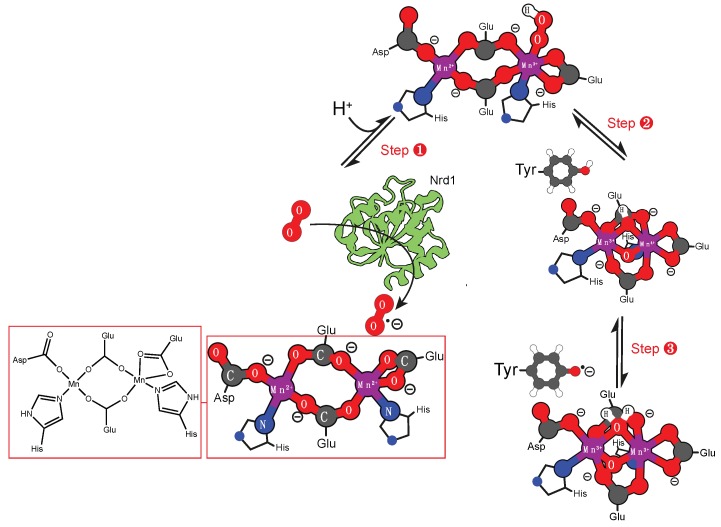
Schematic representation of the catalytic mechanism of RNR from class Ib (Mn–Mn). Regarding amino acid residues belonging to the protein, only the atoms involved in the coordination of the metal were represented to simplify the representation.

The O_2_^−^ molecule then binds to one of the Mn(II) atom, oxidizing it to Mn(III) (Scheme 10—Step ❶). At the same time, one of the oxygens is protonated and an oxo-bridge and a hydroxyl-bridge between the two metal ions is established (Scheme 10—Step ❷). At the end of this process, the metal ions Mn(II)–Mn(II) are oxidized to Mn(III)–Mn(IV). The resulting reaction intermediate is very similar to the one that was observed in the mechanism of class Ia (Scheme 9) [164,165].

In the last step of the catalytic mechanism, a nearby tyrosine residue loses one hydride for the hydroxyl group that is coordinated to the two Mn ions, generating the desired tyrosyl radical (Scheme 10—Step ❸). When the hydride transfer occurs, the Mn(II) is reduced by the hydroxyl molecule, and a water molecule is formed [164,165]. Once these steps are complete, the enzyme is ready for a new turnover.

In contrast to the Fe–Fe center of class Ia, no tryptophan residue is involved in the formation/stabilization of the tyrosyl radical in class Ib [164].

### 4.3. Cobalt Cofactor

The RNRs from class II are oxygen-independent and use a cobalt-containing cobalamin (vitamin B_12_) cofactor. Although many studies have been devoted to understanding the mechanism for the radical formation, it continues to be poorly understood. However, other enzymes employ the same cofactor to generate radicals, from which more information is available and can provide more insights to understand more clearly how these radicals are generated. One of these enzymes is methionine synthase (EC 2.1.1.13, MetH), which catalyzes the regeneration of methionine from homocysteine. This enzyme is a modular protein that contains different domains, which are implicated in the binding and activation of different substrates and cofactors involved in the catalytic cycle.

One of these domains contains a Zn ion that activates the substrate of the enzyme, homocysteine, to a reactive thiolate. The other domain of MetH contains a cobalt-containing cobalamin (vitamin B12) cofactor and is where the catalytic reaction takes place.

Before the cobalt-containing cobalamin (vitamin B_12_) cofactor can react with the activated substrate, it needs to be activated by 5-methyl-tetrahydrofolate (5-methyl-THF) [166,167]. In this process, the methyl group from the 5-methyl-THF becomes coordinated directly to the metal ion and THF is released to the solvent. During this reaction, the metal cofactor is oxidized from Co(I) to Co(III), an active site histidine residue becomes coordinated to the Co(III) ion, and the metal cofactor changes from a tetra-coordinated to a hexa-coordinated configuration [166].

**Scheme 11:**
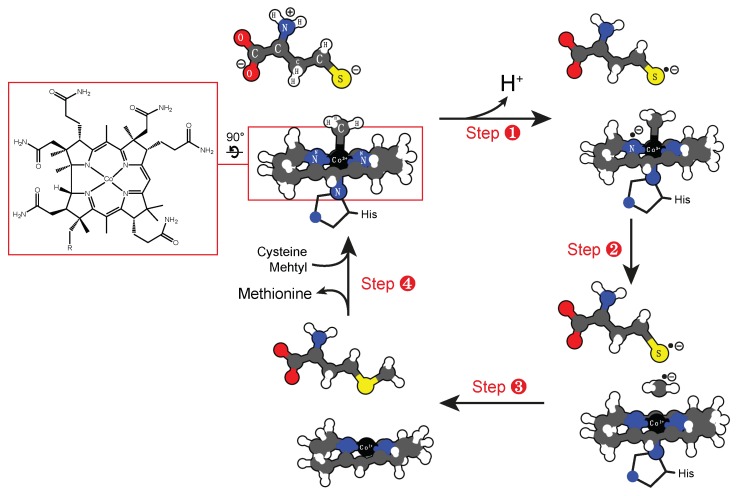
Schematic representation of the catalytic mechanism of methionine synthase (MetH). Regarding amino acid residues belonging to the protein, only the atoms involved in the coordination of the metal were represented to simplify the representation.

The reaction of the substrate with the Co cofactor has been a subject of great debate in the last decade [168,169,170,171,172]. Some authors suggest that it goes through a S_N_2 type of mechanism and involves the nucleophilic attack of the anionic sulfur of the substrate to the carbon of the methyl group that is coordinated to Co ion. In this hypothesis, no radical species are involved in the reaction, and at the end of this process, the Co(III) becomes reduced to Co(I) [168,169,170,171,172,173]. A new hypothesis, however, has been advanced that involves a radical reaction [174] that is in line with recent studies devoted to this enzyme [175]. In this new version of the mechanism, the negatively charged substrate acts as a reductive specie transferring one electron to the Co cofactor, generating two radical species inside the active site (Scheme 11—Step ❶). Subsequently, the electron from the cofactor is transferred to the methyl group, from which results a methyl radical (Scheme 11—Step ❷). The group then binds to the thiol radical of the homocysteine and forms methionine (Scheme 11—Step ❸). At the end of this process, the Co(III) ion is reduced again to Co(I) and the enzyme is ready for a new turnover (Scheme 11—Step ❹) [175,176].

Computational studies estimate an energy barrier for the described reaction of ~8.5 kcal/mol and is comparable to the also theoretically obtained barrier for the S_N_2 reaction pathway hypothesis, which is of 10.5 kcal/mol [173,175,176]. An experimental *k*_cat_ of 27.1 s^−1^ was obtained under specific experimental conditions, in line with the computational studies [177].

The cobalamin cofactor, and in particular the Co ion, has a preponderant role in this mechanism, first promoting the stabilization of the radical specie, and secondly, allowing the stabilization of the methyl radical, which otherwise could be toxic to the cells. 

## 5. Enzymes that Catalyze the Formation of Superoxide

A superoxide is a compound that contains the superoxide anion with the chemical formula O_2_^−^. Superoxide is formed by some enzymes to catalyze some specific reactions. Because superoxide can be very toxic to the cells, these metalloenzymes keep it coordinated to the metal cofactors and therefore preclude their dissociation to the solvent, a condition that could put the cells in danger. Two examples that employ copper and iron cofactors are described in the following sections.

### 5.1. Copper Cofactor

Dopamine β-hydroxylase (EC 1.14.17.1, DBH), also known as dopamine β-monooxygenase, is an enzyme that has an uncommon non-coupled binuclear Cu–Cu cofactor and catalyzes the production of norepinephrine and water through the hydroxylation of the benzyl carbon of dopamine and di-oxygen through a radical mechanism [178].

The crystallographic structure of this enzyme [179] shows one copper atom (Cu1) coordinated to two histidine residues and a methionine residue. The second copper metal ion (Cu2) is coordinated by three histidine residues [180]. Both Cu1 and Cu2 metal ions play a catalytic role in the mechanism.

**Scheme 12:**
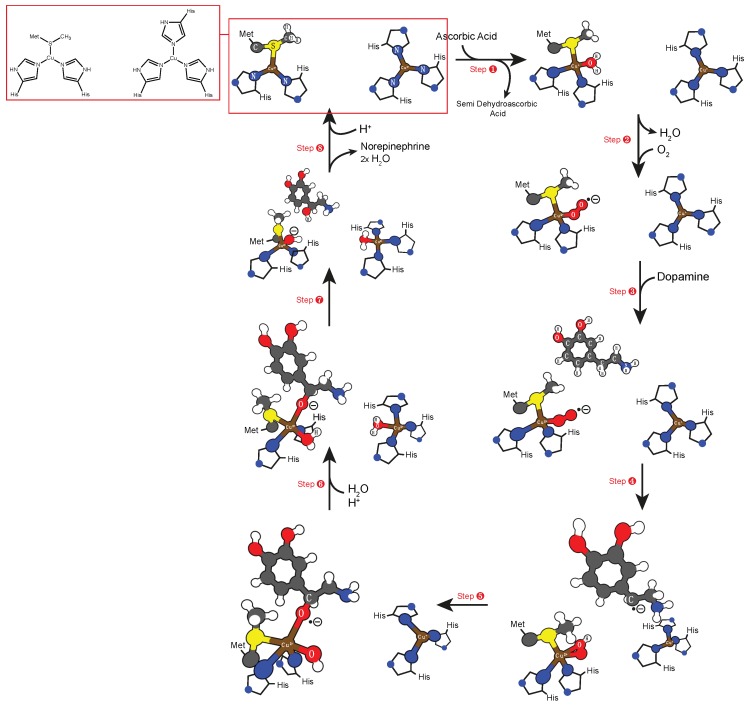
General description of the catalytic mechanism of dopamine β-hydroxylase (DBH). Regarding amino acid residues belonging to the protein, only the atoms involved in the coordination of the metal were represented to simplify the representation.

The first step of the catalytic mechanism starts with the reduction of the two Cu(II) atoms to Cu(I) by two ascorbic acid molecules that become oxidized to semi-dehydroascorbate (Scheme 12—Step ❶) [181]. Afterwards, a di-oxygen molecule binds to Cu1(I), where it is rapidly reduced to O_2_^−^ and Cu1(I) is oxidized back to Cu(II) (Scheme 12—Step ❷).

When substrate is available in the active site (dopamine), it becomes enclosed by a network of hydrogen bonds provided by a neighbor tyrosine and two glutamate residues that ensures the stereospecific of the chemical reaction and moves it close to both cooper metal ions (Scheme 12—Step ❸). Afterwards, the homolytic cleavage of the substrate occurs, and by the action of the activated di-oxygen anion (O_2_^−^), an OOH species is formed that becomes coordinated by both oxygens to Cu1 (Scheme 12—Step ❹). The generated radical located in the carbon of the substrate then attacks the non-protonated oxygen of the OOH species, cleaving the O–O bond and generating an oxo-radical (Scheme 12—Step ❺). At this moment, an electron-transfer between the Cu2 and Cu1 sites, that is not completely understood, takes place. The Cu2 becomes oxidized (Cu(II)) and becomes coordinated to an additional water molecule (Scheme 12—Step ❻). At the same time, the oxo-radical of the substrate undergoes a one-electron reduction, and a proton donor (probably a water molecule) provides a proton that converts the hydroxyl species into a water molecule. The last step of the catalytic mechanism involves the abstraction of a proton from the water molecule coordinated to the Cu1 atom by the negatively charged oxygen atom of the substrate (Scheme 12—Step ❼). Subsequently, the substrate becomes hydroxylated, and it is quickly released from the active site, leaving the enzyme ready for a new catalytic cycle (Scheme 12—Step ❽) [180].

The role played by both Cu ions in the catalytic mechanism of DBH is very distinct but both of them have an active role in the chemical processes that takes place in the active site. The Cu1 is responsible for the initial activation of the dioxygen molecule and the Cu2 is required for the second one-electron reduction. It is worth mentioning that the hydroxylation function of DBH is only possible due to the uncommon non-coupled binuclear Cu–Cu cofactor. By displacing the two metals, the enzyme enables the two-electron oxidation of the substrate through two sequential one-electron steps [180]. This highly controlled oxidation is necessary to prevent side reactions that could oxidize the hydroxyl and amine groups of reaction intermediates.

The experimental *k_cat_* found for the substrate dopamine varies between 13 and 110 s^−1^ depending on the experimental conditions [182,183].

### 5.2. Binuclear Iron Cofactor

The generation of reactive and unstable oxygen species can also be accomplished by enzymes containing iron cofactors. One of these examples is found in the enzyme myo-inosnitol oxygenase (EC 1.13.99.1, MIOX) that catalyzes the oxidation of myo-inositol (MI) to glucuronic acid (GA) [182,183,184].

MIOX employs two catalytic iron metals, (Fe1(II) and Fe2(II)), that are kept together through the simultaneously coordination to a hydroxide molecule. Both iron ions are also coordinated by two histidine residues. The Fe1(II) is also coordinated to a water molecule and an oxygen atom from the carboxylate group of an aspartate residue from the active site.

**Scheme 13:**
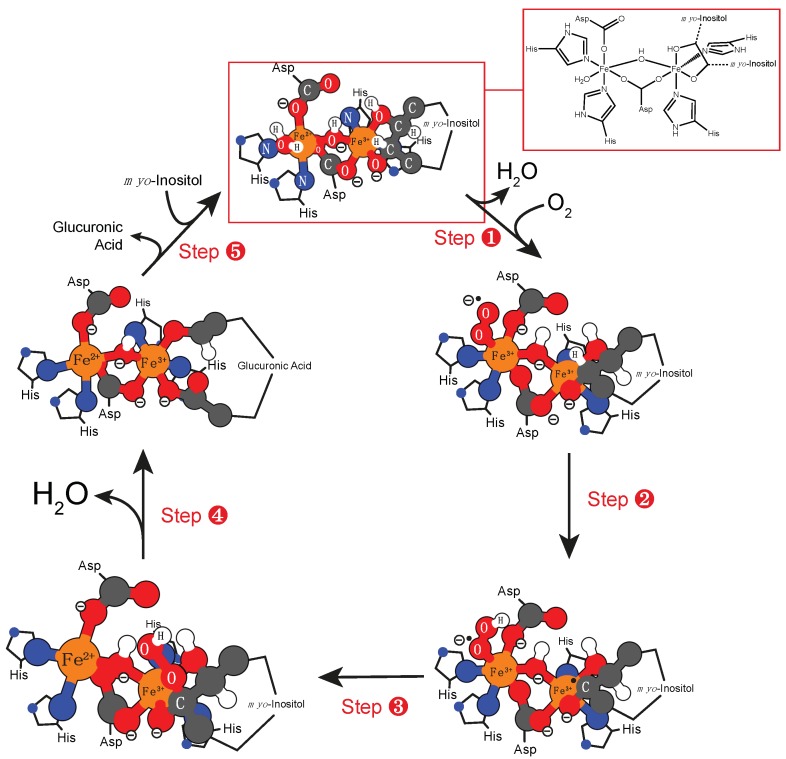
General description of the catalytic mechanism of myo-inosnitol oxygenase (MIOX). Regarding amino acid residues belonging to the protein, only the atoms involved in the coordination of the metal were represented to simplify the representation.

When substrate is available in the active site (MI), it becomes coordinated to the Fe2 ion through two hydroxyl groups. In the first step of the catalytic mechanism (Scheme 13—Step ❶), the water molecule that is coordinated to the Fe1(II) ion is replaced by an oxygen molecule. This molecule is then rapidly reduced to superoxide at the same time that Fe1(II) is oxidized to Fe1(III).

The superoxide molecule remains coordinated to the Fe1(III) ion and in the next step, the hydride transfer from the carbon atom of the MI (Scheme 13—Step ❷) to the superoxide molecule takes place. This step originates a radical intermediate that is stabilized by the Fe2(III) and a hydroperoxide that remains bounded to the Fe1(II) ion. These two species have a short half-life time, reacting quickly with each other in the next step of the catalytic mechanism. In this reaction, the hydroperoxide becomes covalently bonded to the radical intermediate and the generated compound remains coordinated to the Fe2(III) (Scheme 13—Step ❸).

In the next step of the catalytic mechanism (Scheme 13—Step ❹), the hydroxyl group that is coordinated to Fe2(III) is deprotonated by a base, at the same time that the cleavage of the hydroperoxo group and the concomitant cleavage of the C–C bond that originates the final product, GA, takes place [185]. Currently, there are no data that confirms what the base that abstracts the proton during this step is, but Morokuma and co-workers suggested that the hydroxide molecule placed between the two metal ions could play that role [185].

Finally, the GA is released from the active site and the enzymatic turnover takes place with the entrance of a new molecule of MI (Scheme 13—Step ❺) [184,185,186].

According to theoretical data, the rate-limiting step of this mechanism is the breakage of the O–O bond that occurs in step 4, requiring an activation energy of 26.9 kcal/mol [185]. Experimental data show a *k*_cat_ for the substrate myo-inositol of 0.183 s^−1^ [187].

## 6. Conclusions

Nature has developed highly efficient metalloenzymes bearing one or more metal cofactors that can carry out an outstanding range of chemical reactions. Some of the most interesting ones produce highly reactive and unstable species, such as hydroxide anions, hydrides, radical species, and superoxide molecules. The importance of these enzymes is enormous since they have paved the way to the emergence of the life as we know today. The formation of such reaction intermediates does not occur easily under biological conditions and only a rationalized selection of a particular metal ion, coordinated to a very specific group of ligands, allows these reactions to proceed.

The review of the catalytic mechanism of these enzymes highlighted that Zn, Mn, and Ni are involved in the formation of hydroxide as reaction intermediates. Zn is able to produce it through mononuclear metal centers in CA and HDAC8 enzymes, but also through a binuclear Zn–Zn center in the case of LeuAP. This last binuclear center likewise serves as a template to the Mn–Mn center of prolidase enzyme, which catalyzes an analogous reaction through a similar mechanism. Additionally, urease uses a binuclear metal center of Ni to produce a hydroxide intermediate involved in the urea degradation. (Table 1). 

Enzymes like ADH can catalyze the production of hydride intermediates using binuclear Zn–Zn metal centers where one of the metal ions has a structural role and the other one has a catalytic character. The formation of hydride intermediates is also mediated by enzymes as XO and FdH that use Mo as a metal cofactor complexed with one or two pterin molecules. (Table 1). 

In terms of radical formation, RNR enzymes can produce radical species through binuclear Fe–Fe or Mn–Mn centers accordingly if they belong to the Ia or Ib families, respectively. The MetH is an enzyme that uses a Co ion coordinated to a cobalamin molecule and a histidine residue to help the production of a methyl radical intermediate (Table 1).

Finally, MIOX and DBH enzymes use binuclear Fe–Fe and Cu–Cu centers, respectively, to promote the generation of superoxide molecules inside the active site that act as crucial intermediates in the general catalytic mechanism (Table 1).

A very important feature of these metalloenzymes is that the unstable and reactive reaction intermediates that are produced during catalysis remain coordinated to the metal ion until it reacts with another molecule and generates the final product of the reaction. This not only reduces the concentration of these species in the cells, which otherwise can be lethal due to their high toxicity, but also turns the catalytic process very efficient.

Several experimental and computational studies have already unraveled the outstanding biochemistry behind the processes that are catalyzed by these enzymes. In some cases, the same highly reactive and unstable species can be produced by enzymes containing very different metal coordination spheres, different in terms of the identity of the transition metal, number and nature of the ligands involved, and even number of metal atoms. This diversity ensures alternative strategies to generate the same chemical species through rather different mechanisms, illustrating the rich chemistry that nature can offer. Together, these studies have become very important because understanding the enzyme structure and the reaction pathways is a direct way to address the questions about the nature of the enzymatic power and enzyme evolution. This is very important for the development of new catalysts but also to understand how their function can be improved or inactivated. In the latter case, this knowledge can be used to develop new inhibitors for metalloenzymes that are currently drug targets for the treatment of several diseases.

## Figures and Tables

**Table 1:** Summary of the reviewed enzymes organized by their metal cofactor and respective produced intermediate.

	Catalysed Reaction			
Metal	Hydroxide	Hydride	Radical	Superoxide
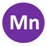	Prolidase		RNR-Ib	
	(Asp)**Mn-**(Asp)(Glu)(OH_2_)-**Mn**(Glu)(His)		(His)(Asp)**Mn**-*(Glu)_2_*-**Mn**(His)(Glu)	
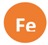			RNR-Ia	MIOX
			(His)(Glu)**Fe**-*(Glu)_2_*-**Fe**(His)(Glu)	(His)_2_(OH_2_)(Asp)**Fe**-*(OH)(Asp)*-**Fe**(His)_2_
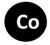			MetH	
			**Co**(Cobalamin)(His)	
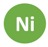	Urease			
	(His)_2_(OH_2_)**Ni-**(Lys*)(OH_2_)-**Ni**(His)_2_(OH_2_)(Asp) *carbamylated			
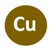				DBH
				(His)_2_(Met)**Cu**--**Cu**(His)_3_
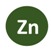	CA			
	**Zn**(His)_3_(OH_2_)			
	HDAC8			
	**Zn**(Asp)_2_(His)(OH_2_)			
	LeuAP	ADH		
	(Asp)**Zn-**(Asp)(Glu)(OH_2_)-**Zn**(Lys)(Asp)	(Cys)_4_**Zn^✸^**--**Zn**(His)(Cys)_2_(OH_2_)		
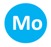		XO		
		**Mo**OS(OH)(Pterin)		
		FdH		
		**Mo**S(Pterin)_2_(Cys)		

**^✸^** structural function.
